# Influence of Halogens on PS_4_^3–^ Speciation and Formation Pathways in Acetonitrile

**DOI:** 10.1021/acs.inorgchem.6c01436

**Published:** 2026-06-06

**Authors:** Zachary Warren, Didem Sürsal, Julián Alexandre Rodríguez Peinado, Nataly Carolina Rosero-Navarro

**Affiliations:** † Instituto de Cerámica y Vidrio−CSIC, C/Kelsen 5, Madrid 28049, Spain; ‡ Universidad Autónoma de Madrid, Ciudad Universitaria de Cantoblanco, Madrid 28049, Spain; § 162339 Sakarya University, Department of Metallurgical & Materials Engineering, Esentepe Campus, Adapazarı 54050, Sakarya, Turkey

## Abstract

Liquid-phase synthesis of Li_2_S–P_2_S_5_ derived sulfide solid electrolytes often proceeds through suspended precursor particles and solvated thiophosphate intermediates, yet analogous intermediate formation during argyrodite Li_6_PS_5_X synthesis remains poorly understood. Here, we investigate halide-modulated thiophosphate speciation in acetonitrile using minute-resolved in situ Raman spectroscopy and ex situ XRD. The PS_4_^3–^-associated Raman feature near 420 cm^–1^ develops progressively faster across the series I^–^ < Br^–^ < Cl^–^, while the ACN-associated band near 390 cm^–1^ evolves concurrently with thiophosphate formation, consistent with changes in the local ACN coordination environment. XRD of recovered powders shows ACN complexed LPS intermediates in the halide-free reaction, predominantly amorphous material for LiI, partial argyrodite formation for LiBr, and near-complete Li_6_PS_5_Cl formation for LiCl. These results suggest that halide identity influences both thiophosphate speciation and phase selection through coupled solubility, interfacial reactions, and Li^+^ coordination effects. The findings provide a mechanistic basis for using halide chemistry to modulate low-temperature liquid-phase synthesis of sulfide solid electrolytes.

## Graphical abstract

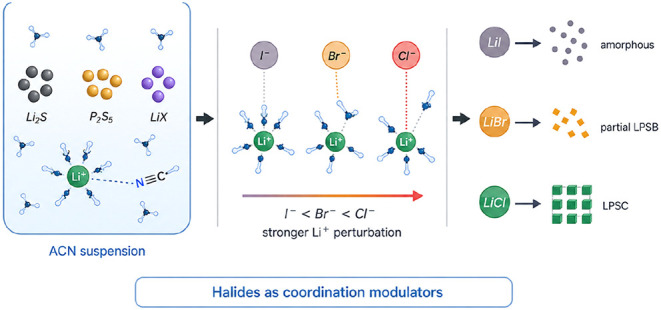

## Introduction

Sulfide solid electrolytes (SSEs) have emerged as critical materials in the development of next-generation solid-state batteries (SSBs), owing to their high ionic conductivity, wide electrochemical stability window, and superior mechanical properties compared to liquid electrolytes. These features make them highly attractive for replacing conventional liquid electrolytes in lithium-ion batteries (LIBs), offering potential solutions for safety concerns, such as leakage, flammability, and dendrite formation, while simultaneously enhancing energy density.

Among the various SSE systems, those based on the Li_2_S–P_2_S_5_ composition have gained significant attention. Li_2_S–P_2_S_5_-based electrolytes exhibit some of the highest reported ionic conductivities, with values as high as 10^–2^ S cm^–1^ at room temperature.^1^ When modified with lithium chloride (LiCl), as in the Li_2_S–P_2_S_5_–LiCl system, the ionic conductivity can reach similarly high levels,^2^ providing robust performance for SSB applications. Additionally, the mechanical properties of SSE electrolytes, characterized by their favorable mechanical moduli,^3^ further strengthen their suitability for battery systems.

To explore more cost-effective and scalable methods of synthesizing these SSEs, solution-based processes have been developed as an alternative to traditional solid-state reactions.^4,5^ In solution-based synthesis, the precursors are reacted in solvents, allowing for a more uniform distribution of elements and facilitating the formation of desired phases at lower temperatures. This approach not only simplifies the synthesis but also enhances the control over the resulting microstructure and ionic transport properties of the electrolyte. Compared to solid-state reactions, which often require high temperatures and prolonged reaction times, solution-based methods offer a more energy-efficient and scalable route to producing high-quality SSEs.

In this work, “liquid-phase synthesis” is used as the general term for reactions carried out in an organic solvent medium. More specifically, the present reactions are best described as ACN-mediated suspension syntheses, because the initial inorganic precursors are not assumed to be fully dissolved. Instead, dispersed Li_2_S/P_2_S_5_/LiX particles coexist with dissolved or solvated thiophosphate species that evolve during reaction. We therefore distinguish between dispersed precursor phases, dissolved/solvated thiophosphate intermediates, and the crystalline phases recovered after solvent removal.

The two most studied sulfide electrolytes synthesized via solution methods are β-Li_3_PS_4_ ^6–9^ and Li_6_PS_5_Cl^10–14^ (argyrodite). β-Li_3_PS_4_ is often synthesized using tetrahydrofuran (THF) or acetonitrile (ACN) as a solvent, which facilitates the reaction between Li_2_S and P_2_S_5_ precursors, leading to the formation of a stable solid electrolyte with moderate to high ionic conductivity (10^–4^ S cm^–1^). On the other hand, Li_6_PS_5_Cl, typically synthesized via a wet-chemical route in ACN or pyridine, has garnered attention due to its higher ionic conductivity (10^–3^ S cm^–1^).^4,5,15^

Both β-Li_3_PS_4_ and Li_6_PS_5_Cl electrolyte systems during synthesis facilitate the formation of tetrahedral thiophosphates (PS_4_^3–^), which contribute distinctively to their overall ionic conductivities. Despite the chemical similarities in structure, there is a noticeable difference in the mechanism of formation of the thiophosphates between β-Li_3_PS_4_ and Li_6_PS_5_Cl that is observable by XRD. β-Li_3_PS_4_ has been shown to form solvent/precursor complexes during solution-based synthesis, which break down at higher temperatures and lead to the crystallization of the β-phase of Li_3_PS_4_.^16^ These solvent/precursor complexes have been observed on XRD patterns in multiple reports of solution-based synthesis of Li_2_S–P_2_S_5_, which generate distinctive XRD patterns.^17–19^ On the contrary, these solvent/precursor complexes have not been reported during solution-based synthesis of Li_6_PS_5_Cl. Instead, the crystal phase of Li_6_PS_5_Cl can easily be synthesized and identified by XRD in samples that are subjected to low temperature annealing after synthesis in solvents.^11^ When high temperature is applied to the Li_6_PS_5_Cl SSEs, the electrochemical properties are enhanced, however there is merely a minimal change in phases present on the XRD patterns.^20^ This observation suggests that the mechanism of formation of PS_4_^3–^ in Li_6_PS_5_Cl during solution-based synthesis is dissimilar to the mechanism of PS_4_^3–^ in β-Li_3_PS_4_. The effect that halogens have on the structure of Li_6_PS_5_Cl has been reported from solid-state reactions and several solution-based synthesis reactions alike, however the mechanisms of formation for the phases of Li_6_PS_5_Cl in solution have yet to be elucidated.

To better understand the reaction mechanisms and formation pathways in solution-based synthesis, Raman spectroscopy has proven to be a powerful tool. By monitoring the vibrational modes of the PS_4_^3–^ units in real-time, insights into the structural evolution of SSEs like Li_6_PS_5_Cl during synthesis may become more evident. When coupled with complementary structural techniques such as X-ray diffraction (XRD), Raman spectroscopy provides a direct correlation between molecular-level thiophosphate speciation and long-range crystalline phase evolution during synthesis.

This study aims to build on the current understanding of solution-based sulfide electrolyte synthesis, focusing on the formation mechanisms and properties of Li_6_PS_5_X, and the interactive effects that halogens contribute during synthesis. A deeper understanding of the effects that halogens contribute to sulfide electrolytes can enable the development of tailored SSEs and promote broader implications of solution processing for scalable, high-performance SSE production.

## Experimental Methods

All reagents used in this study, including lithium sulfide (Li_2_S, 99.9% purity, Aldrich), phosphorus pentasulfide (P_2_S_5_, 99.9% purity, Aldrich), lithium chloride (LiCl, 99.9% purity, Aldrich), lithium bromide (Aldrich, 99.9%), and lithium iodide (Aldrich, 99.9%) were handled inside an argon-filled glovebox (O_2_ < 0.5 ppm, H_2_O < 0.5 ppm) to prevent contamination from moisture or air. Acetonitrile (ACN, LiChrosolv, Supelco) was used as the solvent for all reactions. Because the objective was to compare halide identity under fixed reaction conditions, concentration dependence was not systematically varied in the present study.

ACN was selected as a controlled model solvent because it is widely used in liquid-phase Li_2_S–P_2_S_5_ chemistry, is polar aprotic, has sufficient polarity to promote thiophosphate speciation, and can be removed under relatively mild conditions. The purpose of the present study is not to compare solvent families, but to isolate the effect of halide identity under a fixed solvent environment. Holding ACN constant allows the observed differences among LiCl, LiBr, and LiI to be interpreted primarily in terms of halide-modulated coordination and interfacial reaction effects.

The synthesis of SSEs was performed using microwave-assisted heating (MW400, Anton Paar), coupled with in situ Raman spectroscopy (Cora 5001, Anton Paar, 785 nm laser) to monitor the reaction progress. The five reaction systems investigated were 3Li_2_S–P_2_S_5_, 5Li_2_S–P_2_S_5_-2LiCl, 5Li_2_S–P_2_S_5_-2LiBr, 5Li_2_S–P_2_S_5_-2LiI, and 3Li_2_S–P_2_S_5_-0.1LiCl. The P_2_S_5_ concentration was fixed at 200 mM for all reactions to maintain sufficient Raman signal intensity while preserving manageable slurry viscosity and comparable precursor loading across the halide series. This fixed-concentration design allows direct comparison of halide identity under otherwise equivalent reaction conditions. The molar ratios and calculated nominal concentrations of Li_2_S, P_2_S_5_, and LiX are summarized in Table S1 of the Supporting Information. A full concentration-dependence study was not pursued because the objective of the present work was to isolate halide-modulated pathway differences under a constant ACN reaction environment. The reactions were carried out in ACN at 70 °C for 3 h. During the reaction, Raman spectra were collected in situ every minute using a 785 nm laser with a power of 450 mW and an exposure time of 1 s per acquisition.

After completion of the reaction, bulk solvent was removed using a rotary evaporator at 180 rpm, 150 mbar, and 50 °C for 3 h. The resulting powders are referred to here as solvent-removed products. This treatment removes bulk ACN under the stated conditions but does not imply quantitative removal of all coordinated or strongly retained ACN.

To complement the Raman results, X-ray diffraction (XRD) analysis was performed with Cu kα radiation for phase identification and confirmation of the crystallinity of the samples, specifically targeting the presence of β-Li_3_PS_4_, Li_6_PS_5_Cl, and solvent/precursor complexes.

## Results and Discussion

The effect of lithium halide salts on the solution-phase synthesis of lithium thiophosphate electrolytes was first evaluated using in situ Raman spectroscopy during reaction of Li_2_S and P_2_S_5_ in acetonitrile (ACN) at 70 °C. Figure 1 summarizes the time-resolved evolution of the reaction without halide and in the presence of LiI, LiBr, and LiCl. In the undoped LPS system (Figure 1a–c), gradual development of the band near 420 cm^–1^, assigned to the symmetric stretching mode of PS_4_^3–^ tetrahedra, indicates progressive thiophosphate formation over the 3 h reaction. The waterfall plot and corresponding heatmap show slow spectral evolution, and integration of the 420 cm^–1^ peak confirms delayed conversion kinetics under these conditions. Addition of LiI (Figure 1d–f) produces minimal change relative to undoped LPS, with similar onset and growth of the PS_4_^3–^ associated Raman band. In contrast, LiBr (Figure 1g–i) leads to earlier development of the PS_4_^3–^ band, while LiCl (Figure 1j–l) exhibits the most rapid and pronounced increase in intensity. The systematic trend-LiI < LiBr < LiCl in reaction rate—correlates inversely with halide ionic radius and directly with charge density, indicating that halide identity exerts a strong influence on early stage thiophosphate formation.

**Figure 1:**
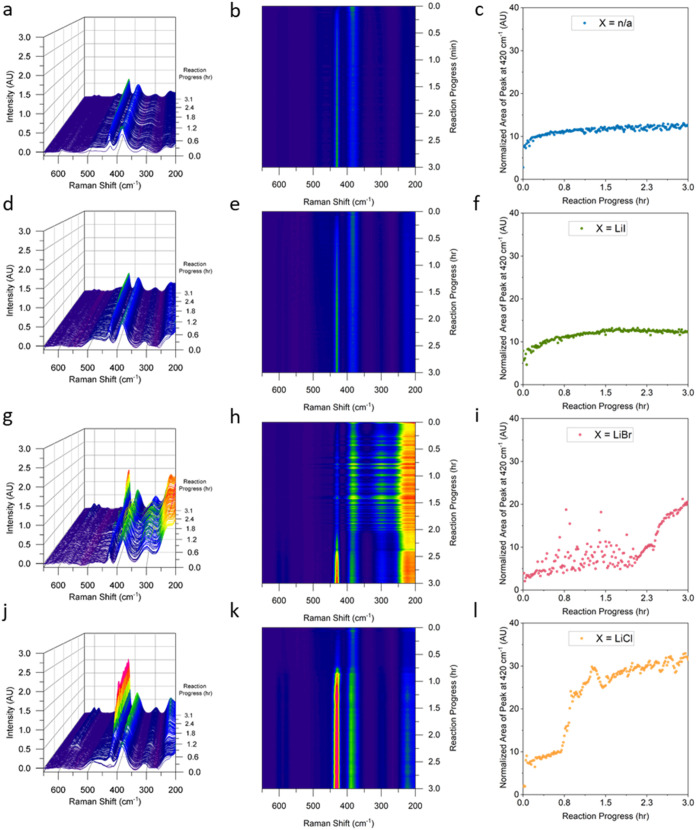
In situ Raman investigation of halide-modulated LPS formation in acetonitrile at 70 °C over 3 h. (a–c) Undoped LPS: (a) waterfall plot, (b) heatmap, and (c) integrated intensity of the 420 cm^–1^ PS_4_^3–^ band versus time. (d–f) LiI-containing reaction. (g–i) LiBr-containing reaction. (j–l) LiCl-containing reaction. Increasing halide charge density (I^–^ → Br^–^ → Cl^–^) accelerates PS_4_^3–^ formation.

In addition to the PS_4_^3–^ associated feature near 420 cm^–1^, the spectra show an ACN-associated band near 390 cm^–1^.^21^ The concurrent increase of this ACN band with the PS_4_^3–^ signal suggests that thiophosphate formation is accompanied by changes in the local ACN coordination environment. In particular, the stronger relative ACN contribution observed in the chloride-containing reaction is consistent with disruption of persistent Li^+^-ACN coordination, increasing the population or Raman contribution of free or weakly coordinated ACN within the reaction mixture. Because Raman intensity in a reacting suspension can also be influenced by optical scattering and particle dispersion, this feature is interpreted qualitatively as evidence for changes in solvent coordination rather than as a quantitative measure of ACN concentration.

The observed trend is consistent with a coupled solubility–coordination effect rather than HSAB theory alone. Lithium ions are hard Lewis acids, and chloride is the hardest and highest charge-density halide examined. For lithium halides in organic solvents, solubility is often favored as the halide becomes larger and more polarizable, because lower-charge-density anions such as Br^–^ and I^–^ can better stabilize ionic interactions in weakly coordinating media.^22^ Therefore, the accelerated thiophosphate formation observed for LiCl is unlikely to arise simply from a higher concentration of dissolved LiX species. Instead, we propose that chloride modifies the local Li^+^ coordination environment at the solid–liquid interface and within emerging solvated thiophosphate clusters, weakening persistent Li^+^-ACN coordination. Bromide produces an intermediate effect, whereas iodide, despite its expected greater compatibility with organic media, is softer and more polarizable and therefore less effective at perturbing the hard Li^+^ coordination environment.

The structural consequences of these halide-modulated kinetic effects are reflected in the powder X-ray diffraction patterns shown in Figure 2, obtained from the same reactions monitored by Raman spectroscopy. The undoped LPS system (Figure 2a) exhibits diffraction features consistent with an ACN-complexed thiophosphate intermediate, in agreement with prior reports of solvent-coordinated LPS species formed during solution synthesis.^17,23^ The LiI-containing system (Figure 2b) remains largely amorphous, consistent with the limited kinetic enhancement observed in Raman spectra. In contrast, LiBr (Figure 2c) shows partial formation of the argyrodite-type LPSB phase, indicating that intermediate halide hardness partially shifts the reaction pathway toward direct electrolyte formation. Most strikingly, the LiCl-containing system (Figure 2d) displays near-complete formation of LPSC with only minor residual Li_2_S, as expected from stoichiometric considerations. The progression from solvent-complexed intermediates to crystalline electrolyte phases is consistent with the coupled solubility–coordination trend observed by Raman spectroscopy.

**Figure 2:**
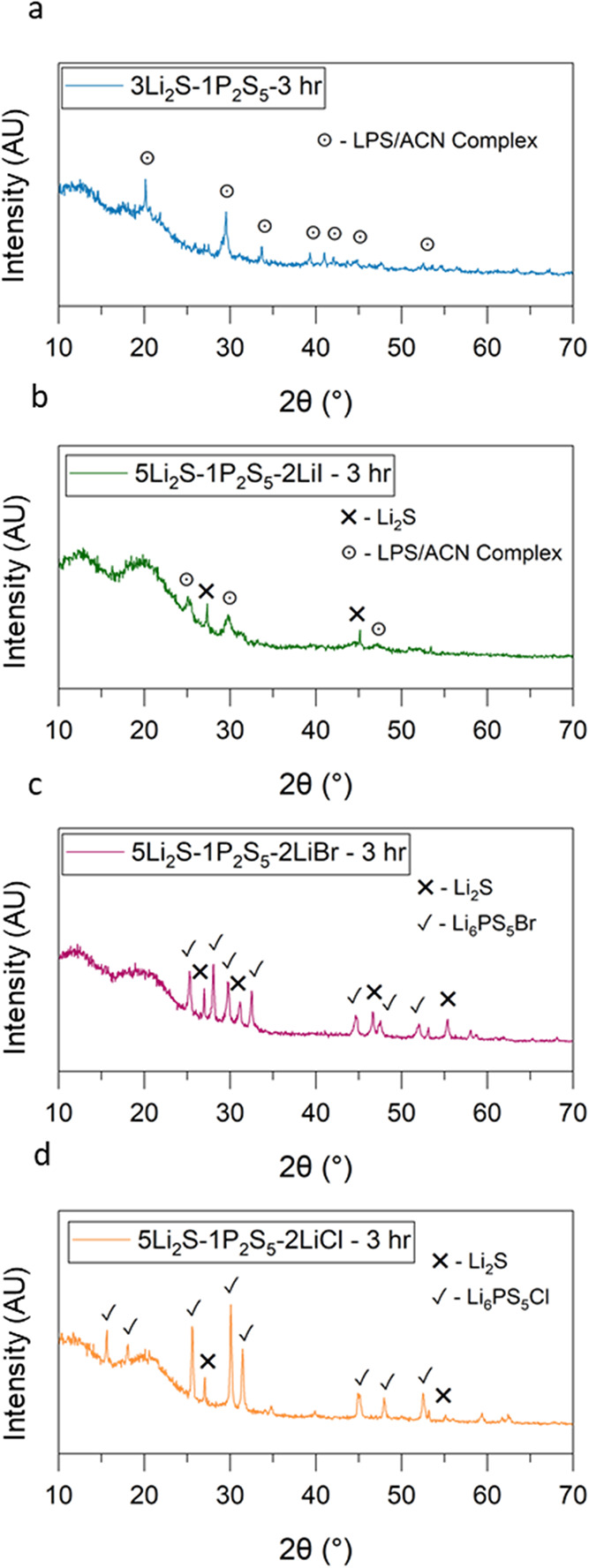
Powder XRD patterns of halide-modified LPS reactions. (a) Undoped LPS showing ACN-complexed intermediate. (b) LiI-containing reaction, predominantly amorphous. (c) LiBr-containing reaction showing partial formation of LPSB. (d) LiCl-containing reaction exhibiting near-complete formation of LPSC with minor residual Li_2_S. Reference reflections for Li_2_S, LiX, β-Li_3_PS_4_, and argyrodite Li_6_PS_5_X are indicated where applicable. Peaks that overlap between Li_2_S/LiX and argyrodite reflections are marked cautiously as Li_2_S.

Because LiX precipitation is a plausible competing pathway during solvent removal, the XRD patterns were also compared with reference patterns of LiCl, LiBr, and LiI (Figure S1). No dominant crystalline LiX phase is observed in the LiCl-containing sample, although overlap between LiX/Li_2_S and argyrodite reflections prevents complete exclusion of minor crystalline halide-containing residues. Therefore, the XRD data support predominant LPSC formation in the chloride-containing reaction, but they do not independently quantify residual LiX content. This distinction is important because the present study focuses on halide-modulated reaction pathways rather than quantitative phase refinement of all residual crystalline or amorphous components.

Time-resolved ex situ XRD of the LiCl-containing reaction further clarifies phase evolution (Figure 3). After 30 min (Figure 3a), the pattern is dominated by Li_2_S, reflecting incomplete conversion at early stages. After 6 h (Figure 3b), well-defined reflections characteristic of LPSC emerge, indicating successful electrolyte formation. However, after 24 h (Figure 3c), a marked reduction in diffraction intensity is observed, consistent with structural degradation under prolonged exposure to reactive solution conditions. These results reveal a kinetic window in which chloride-assisted coordination control enables rapid electrolyte formation, but excessive reaction time compromises structural integrity, in agreement with our previous observations on sulfide electrolyte stability.^24^

**Figure 3:**
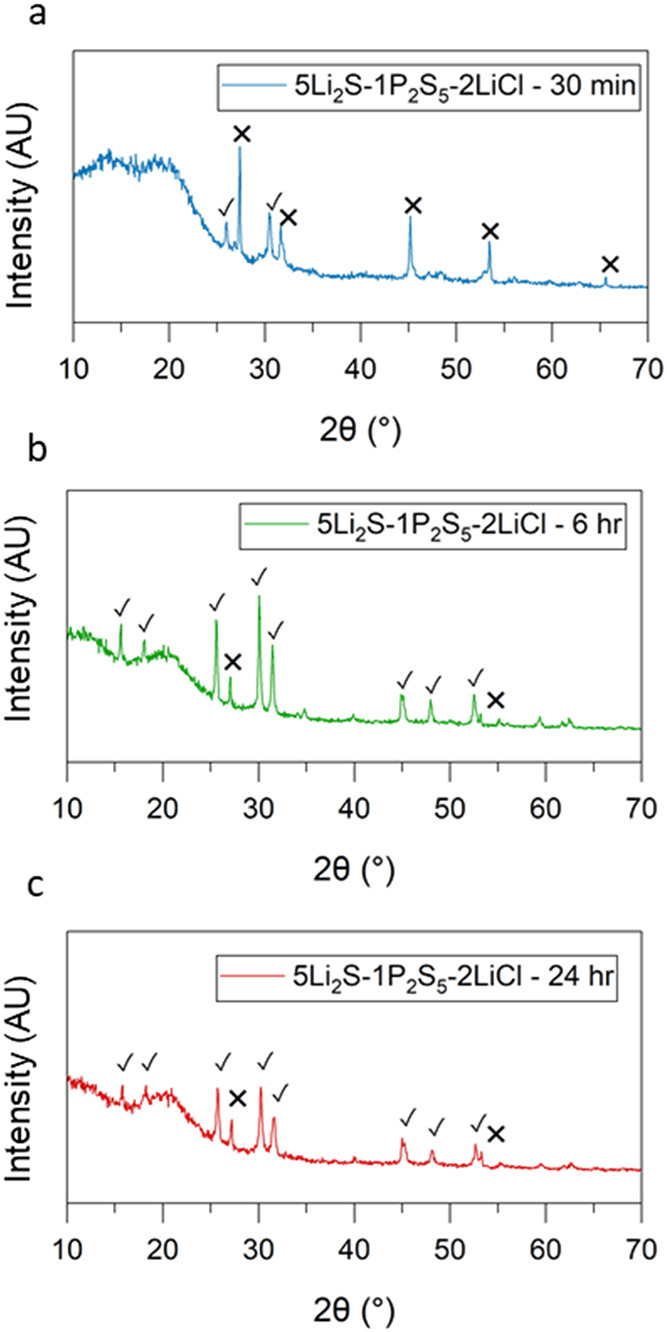
Ex situ XRD of LPSC synthesized with LiCl at different reaction times. (a) 30 min, Li_2_S-dominated. (b) 6 h, well-defined LPSC reflections. (c) 24 h, reduced diffraction intensity indicating degradation under prolonged reaction conditions.

To directly probe whether chloride acts through coordination modulation rather than purely through stoichiometric incorporation, we monitored the 3Li_2_S–P_2_S_5_ reaction containing only 0.1 equiv LiCl relative to P_2_S_5_, corresponding to 20 mM LiCl under the present reaction conditions (Table S1, Supporting Information), by in situ Raman spectroscopy (Figure 4). The waterfall spectra and integrated 420 cm^–1^ PS_4_^3–^ intensity show onset of thiophosphate formation after approximately 2 h, unlike the earlier undoped LPS system despite the low chloride concentration. This result demonstrates that even small amounts of chloride are sufficient to perturb the Li^+^ coordination environment and accelerate PS_4_^3–^ rich thiophosphate formation. The effect is therefore not solely a compositional consequence of argyrodite formation but a coordination-mediated modification of the solution-phase reaction pathway.

**Figure 4:**
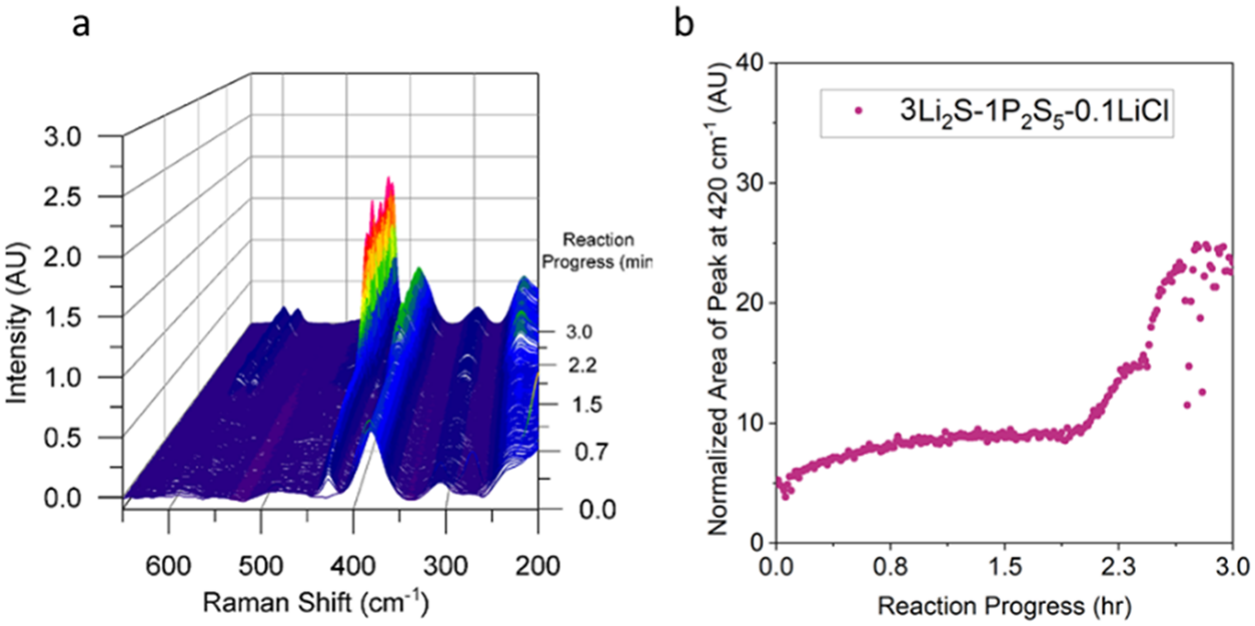
In situ Raman monitoring of the 3Li_2_S–P_2_S_5_ reaction containing 0.1 equiv LiCl relative to P_2_S_5_, corresponding to 20 mM LiCl, in acetonitrile at 70 °C. (a) Waterfall spectra and (b) integrated intensity of the 420 cm^–1^ PS_4_^3–^-associated band reveal accelerated thiophosphate formation relative to undoped LPS.

Further structural evidence of chloride-mediated solvent disruption is provided in Figure 5. The undoped LPS reaction (Figure 5a) clearly exhibits diffraction features corresponding to the ACN-complexed intermediate phase. In contrast, when 0.1 equiv LiCl relative to P_2_S_5_ was added, the reaction yielded β-Li_3_PS_4_ directly without detectable crystalline ACN-complexed reflections. This finding indicates that chloride competitively destabilizes the Li^+^-ACN coordination environment, suppressing formation of detectable crystalline ACN-complexed intermediates that typically requires subsequent thermal decomposition. Consistent with HSAB considerations, the harder chloride appears to perturb the Li^+^-ACN coordination environment more effectively than bromide or iodide, promoting direct β-Li_3_PS_4_ formation under these conditions.

**Figure 5:**
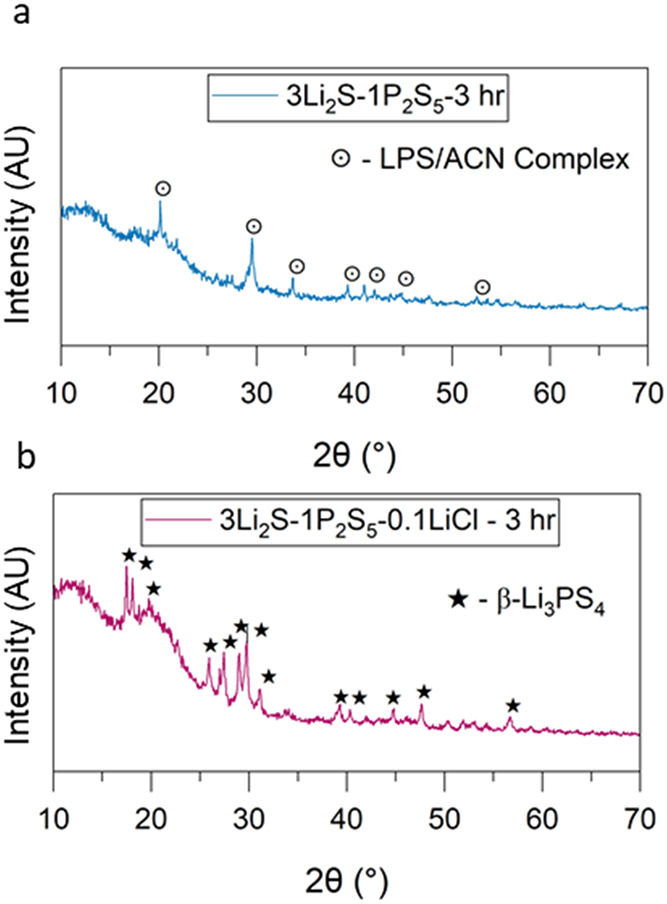
XRD comparison of solvent complexation and chloride-modulated β-Li_3_PS_4_ formation. (a) Undoped LPS showing an ACN-complexed intermediate phase. (b) Reaction containing 0.1 equiv LiCl relative to P_2_S_5_, corresponding to 20 mM LiCl, yielding β-Li_3_PS_4_ without detectable crystalline ACN-complexed reflections.

The absence of detectable crystalline ACN-complexed reflections should therefore be interpreted as suppression of long-range ordered solvent-complexed phases, not as quantitative evidence for complete ACN removal.

Figure 6 summarizes the proposed halide-modulated pathway for ACN-mediated thiophosphate formation. The initial reaction mixture is best described as a suspension in which Li_2_S, P_2_S_5_, and LiX phases coexist with solvated or interfacial species, including Li^+^-ACN coordination environments and emerging thiophosphate clusters (Figure 6a). In this environment, ACN coordinates Li^+^ through the nitrile nitrogen, while the added halide perturbs the local Li^+^ coordination environment to different extents. As illustrated in Figure 6b, iodide produces only weak perturbation and largely preserves persistent Li^+^-ACN coordination, bromide produces an intermediate effect, and chloride produces the strongest local perturbation. This trend is consistent with the Raman-observed acceleration of PS_4_^3–^ formation and the XRD-observed progression in recovered phases (Figure 6c), from amorphous or weakly crystalline material for LiI, to partial argyrodite formation for LiBr, and near-complete LPSC formation for LiCl. Therefore, chloride is proposed to act not merely as a final structural component, but as a coordination-modulating species that promotes Li–S reorganization and shifts the ACN-mediated reaction pathway toward crystalline thiophosphate products.

**Figure 6:**
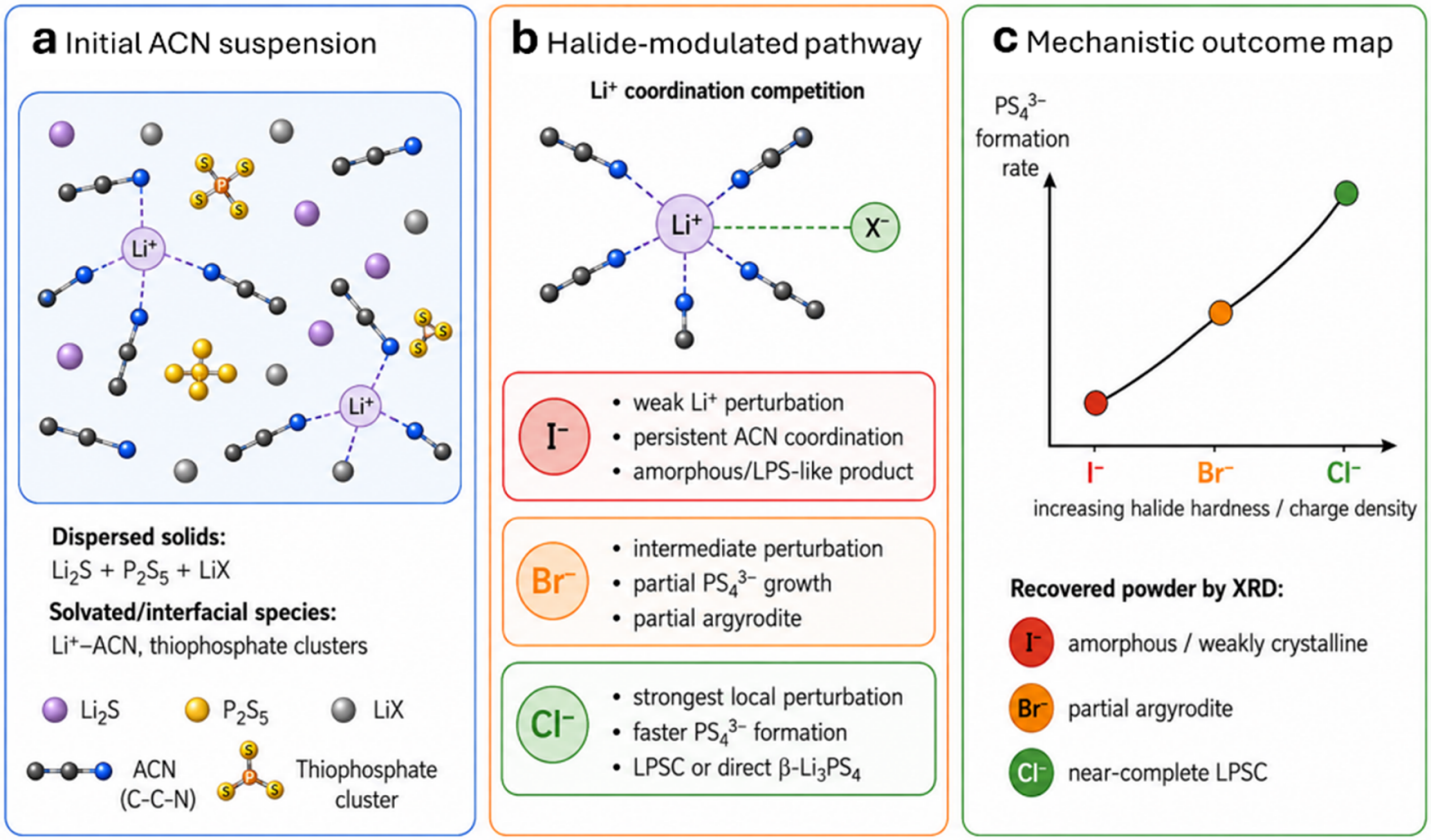
Proposed halide-modulated pathway for ACN-mediated thiophosphate formation. (a) Schematic representation of the initial ACN-mediated reaction mixture, best described as a suspension in which dispersed Li_2_S, P_2_S_5_, and LiX phases coexist with solvated/interfacial species, including Li^+^-ACN coordination environments and thiophosphate clusters. In these coordination motifs, ACN binds Li^+^ through the nitrile nitrogen atom of the linear C–C–N molecule. (b) Proposed halide-modulated pathway showing how halide identity perturbs Li^+^ coordination competition in ACN. Iodide produces weak perturbation and largely preserves persistent ACN coordination, bromide produces an intermediate effect, and chloride produces the strongest local perturbation, promoting faster formation of PS_4_^3–^-rich thiophosphate species. (c) Mechanistic outcome map summarizing the observed trend in PS_4_^3–^ formation rate and the corresponding recovered phases by XRD, progressing from predominantly amorphous or weakly crystalline material for LiI, to partial argyrodite formation for LiBr, and near-complete LPSC formation for LiCl. This schematic emphasizes that halides modulate the ACN-mediated reaction pathway through coordination effects rather than acting solely as final structural components.

## Conclusion

Collectively, these results demonstrate that halide hardness modulates both the kinetics and structural outcome of solution-based thiophosphate synthesis in acetonitrile. A clear and systematic trend emerges across the halide series (I^–^ < Br^–^ < Cl^–^), in which increasing anion charge density accelerates PS_4_^3–^ formation, suppresses detectable solvent-complexed intermediates, and promotes direct formation of crystalline electrolyte phases. The convergence of in situ Raman kinetics and ex situ structural analysis establishes that halide identity does not simply influence final stoichiometry but actively regulates the reaction pathway at the solution-coordination level.

This behavior is consistent with hard–soft acid–base (HSAB) considerations. Lithium ions behave as hard Lewis acids whose coordination environment is sensitive to the hardness of competing bases. Chloride, as the hardest halide examined, perturbs the Li^+^ solvation shell more effectively than bromide or iodide, weakening Li^+^-ACN interactions and facilitating Li–S reorganization. In contrast, iodide, being softer and more polarizable, exerts minimal disruption of the Li^+^ coordination sphere and yields reaction kinetics and structural outcomes comparable to undoped LPS. The intermediate behavior of bromide further supports this interpretation. Importantly, even small concentrations of LiCl are sufficient to accelerate PS_4_^3–^ formation and eliminate solvent-complexed intermediates, supporting a major coordination-mediated contribution rather than a purely compositional effect.

The ability of chloride to destabilize the ACN-complexed LPS intermediate and enable direct formation of β-Li_3_PS_4_ or LPSC can reduce or bypass the need for subsequent thermal decomposition steps under the present conditions. This finding has direct implications for scalable processing. By deliberately tuning competitive coordination interactions in solution, it becomes possible to reduce energy input, simplify synthetic workflows, and potentially enhance phase selectivity without altering the fundamental Li_2_S–P_2_S_5_ stoichiometry.

More broadly, this work establishes halide selection as a mechanistic lever in solution-phase solid electrolyte synthesis. Rather than serving solely as dopants or structural modifiers in the final material, halides can function as coordination modulators that shape reaction kinetics at early stages of formation. This perspective suggests that rational manipulation of Lewis acid–base interactions offers a general strategy for controlling solvent-mediated synthesis routes in sulfide solid electrolytes and related systems.

Future studies may extend this coordination-controlled approach to alternative solvents, mixed-halide systems, or other multicomponent sulfide chemistries, where competitive donor interactions could be exploited to further tailor reaction pathways and materials properties. By integrating coordination chemistry principles into electrolyte synthesis design, new opportunities emerge for lower-temperature, energy-efficient, and industrially scalable production of next-generation solid-state battery materials.

## Supplementary Material



## Notes

LiX reference spectra and impedance analysis of dried LPS and LPSC electrolytes (PDF)

## Abbreviations

LPSX: Li_6_PS_5_X (X = Cl, Br, I)
LPS: Li_2_S–P_2_S_5_
ACN: acetonitrile
HSAB: hard–soft/acid–base
THF: tetrahydrofuran
XRD: X-ray diffraction
