# Supplementary material for: Influence of Halogens on PS4 3– Speciation and Formation Pathways in Acetonitrile
Source: Inorg Chem. 2026 Jun 6;65(24):13504–11. doi: 10.1021/acs.inorgchem.6c01436 (PMC13292203; doi:10.1021/acs.inorgchem.6c01436)
Supplement: Supplementary file 1 [file ic6c01436_si_001.pdf]

Supporting Information

Influence of Halogens on PS_4^{3-} Speciation and Formation Pathways in Acetonitrile

Zachary Warren[1,2], *Didem Sürsal*[1,3], *Julian Alexandre Rodriguez Peinado*[1,2], *Nataly Carolina Rosero-Navarro**[1]

[1]Instituto de Cerámica y Vidrio– CSIC, C/Kelsen 5, Madrid, Spain, 28049

[2]Universidad Autónoma de Madrid, Ciudad Universitaria de Cantoblanco, 28049 Madrid

[3]Sakarya University, Department of Metallurgical & Materials Engineering, Esentepe Campus, 54050, Sakarya, Turkey

Table S1. Reaction mixtures, molar ratios, and nominal component concentrations used in this study.

System	Nominal composition	[P_2S_5]	[Li_2S]	[LiX]	Molar ratio
LPS	$3Li_2S$–P_2S_5	200 mM	600 mM	—	3:1
LPSC	$5Li_2S$–P_2S_5–2LiCl	200 mM	1000 mM	400 mM	5:1:2
LPSB	$5Li_2S$–P_2S_5–2LiBr	200 mM	1000 mM	400 mM	5:1:2
LPSI	$5Li_2S$–P_2S_5–2LiI	200 mM	1000 mM	400 mM	5:1:2
dilute LiCl	$3Li_2S$–P_2S_5-0.1LiCl	200 mM	600 mM	20 mM	3:1:0.1

Table S2. Summary of ionic conductivities

Electrolyte	σ_{Li_25C} (S cm^{-1})
$3Li_2S$-$1P_2S_5$	6.6×10^{-9}
$3Li_2S$-$1P_2S_5$-0.1LiCl	4.5×10^{-6}

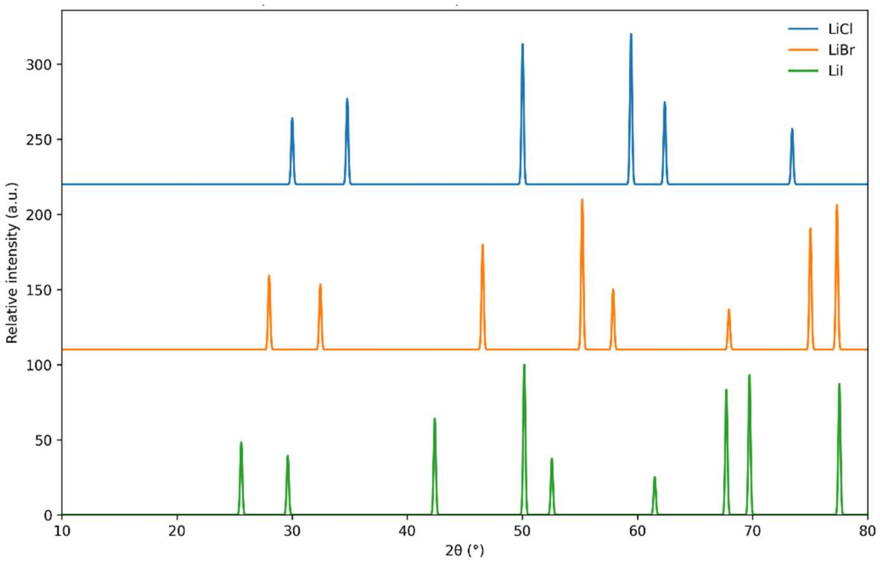

Figure S1. Simulated powder XRD reference patterns of LiCl, LiBr, and LiI generated from the supplied CIF files using Cu Kα radiation (λ = 1.5406 Å). The patterns are vertically offset for clarity. These reference patterns were used to compare possible residual LiX reflections during phase assignment of the reaction products.

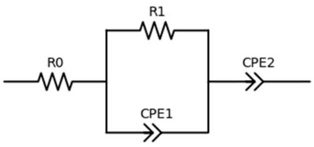

Figure S2. Equivalent circuit model (ECM) for sulfide solid electrolytes, where *R0* is the bulk ionic impedance and *R1* is the grain-boundary impedance. *CPE1* corresponds to the capacitive behavior of the grain boundary and *CPE2* corresponds to mass-diffusion and/or polarization (commonly observed in ion-blocking electrodes).

Electrochemical impedance spectroscopy was performed on the 2 electrolytes synthesized during the reactions: $3Li_2S$-$1P_2S_5$ and $3Li_2S$-$1P_2S_5$-0.1LiCl. Figure S2 illustrates the common equivalent circuit applied to sulfide solid electrolytes

The electrolytes were cold-pressed at 350 MPa at 25 °C for 3 mins. The pellets were sealed in PEEK cells between stainless steel current collectors of 10mm diameter. Impedance measurements were performed at 25 °C between 600 kHz-1Hz with a perturbation of 20 mV.

The $3Li_2S$-$1P_2S_5$ electrolyte exhibited high grain-boundary impedance commonly observed from liquid-synthesis of LPS (figure S3), while the $3Li_2S$-$1P_2S_5$-0.1LiCl exhibited little to no observable grain boundary on the Nyquist (figure S4). Although these ECM fits explain the physical processes withing the electrolyte, the $3Li_2S$-$1P_2S_5$-0.1LiCl electrolyte exhibits > 5% error in the high frequency range with a negative bias. Therefore, the ionic conductivities reported here naturally have a higher-uncertainty (> 5%) and should be used for qualitatively determining the electrochemical differences between the electrolytes.

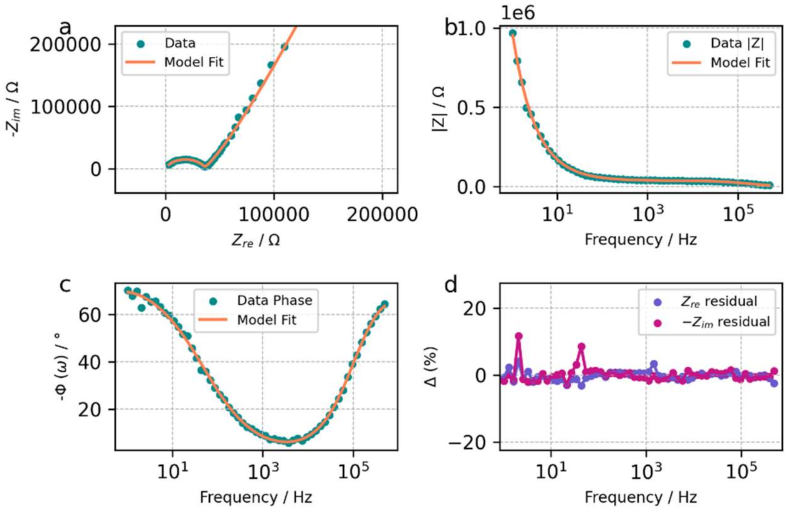

Figure S3. Equivalent-circuit fitting of the impedance spectrum for 3Li□S–P□S□ synthesized in acetonitrile at 70 °C for 3 h and subsequently dried at 50 °C, corresponding to Figure 5a in the main text. The experimental data and fitted model are shown as: **(a)** Nyquist plot, **(b)** impedance magnitude |Z| as a function of frequency, **(c)** phase angle as a function of frequency, and **(d)** residuals of the real and imaginary impedance components. The close agreement between the measured data and model fit supports the suitability of the selected equivalent circuit for describing the impedance response of the sample.

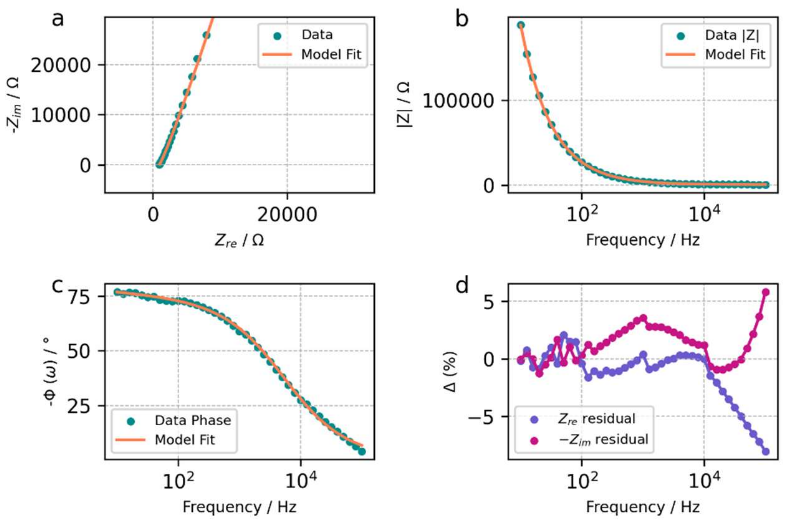

Figure S4. Equivalent-circuit fitting of the impedance spectrum for 3Li□S–P□S□–0.1LiCl synthesized in acetonitrile at 70 °C for 3 h and subsequently dried at 50 °C, corresponding to Figure 5b in the main text. The fitted model is shown together with the experimental data as: **(a)** Nyquist plot, **(b)** impedance magnitude |Z| as a function of frequency, **(c)** phase angle as a function of frequency, and **(d)** residuals of the real and imaginary impedance components. The close agreement between the experimental data and model fit confirms the suitability of the selected equivalent circuit for describing the impedance response of the sample

Ionic conductivity is calculated from the extracted *R*s from the ECMs and with the dimensions of the electrolyte using equation S1.

$$\sigma = \frac{l}{A \times R},$$

Equation S1. Ionic conductivity from R (impedance)

where l is the thickness of the electrolyte, A is the area, and R is the total impedance measured from the ECM.
